# Workplace exposure to asbestos and the risk of kidney cancer in Canadian men

**DOI:** 10.17269/s41997-018-0095-9

**Published:** 2018-09-17

**Authors:** Cheryl E. Peters, Marie-Élise Parent, Shelley A Harris, Linda Kachuri, Lidija Latifovic, Laura Bogaert, Paul J Villeneuve, Canadian Cancer Registries Epidemiology Group

**Affiliations:** 1 Department of Health Sciences, Carleton University, 3rd Floor, Health Sciences Building, Ottawa, ON K1S 5B6 Canada; 2 Institut Armand Frappier-Institut National de la Recherche Scientifique, Laval, QC Canada; 3 Cancer Care Ontario, Toronto, ON Canada; 4 Dalla Lana School of Public Health, University of Toronto, Toronto, ON Canada

**Keywords:** Kidney cancer, Asbestos exposure, Workplace exposure, Tumeurs du rein, Exposition à l’amiante, Exposition professionnelle

## Abstract

**Objective:**

Previous studies considered the role of occupational causes in kidney cancer but were limited by small sample sizes and imprecise exposure assessment. This study examined the relationship between occupational exposure to asbestos and the risk of kidney cancer across a range of jobs in a large, population-based case-control study in Canada.

**Methods:**

Data were from the case-control component of the National Enhanced Cancer Surveillance System, a study conducted between 1994 and 1997 in eight Canadian provinces. Male kidney cancer cases, histologically confirmed, and controls completed questionnaires on socio-demographics, anthropometry, diet, smoking, secondhand smoke exposure, and physical activity. Occupational histories were also collected, including each job held for at least 1 year since the age of 18. Occupational hygienists, blinded to case status, assigned exposure to asbestos, considering intensity, frequency, and probability of exposure (each 3-point scales). Logistic regression was used to estimate the odds of kidney cancer in exposed participants (defined using three metrics) compared to those without asbestos exposure.

**Results:**

There were 712 cases and 2454 controls in these analyses. Ever-exposure to asbestos was associated with 20% increased odds of kidney cancer compared to unexposed workers (OR 1.2, 95% confidence interval 1.0–1.4 when including possibly exposed workers). A small increase in risk was observed with cumulative exposure, while increasing intensity of exposure was related to increased odds of kidney cancer.

**Conclusions:**

This study found some evidence for an association between occupational exposure to asbestos and kidney cancer. Higher intensity of exposure to asbestos had the strongest relationship with kidney cancer risk.

## Résumé

**Objectif:**

Le rôle des causes professionnelles dans le cancer du rein est abordé dans des études antérieures, mais celles-ci sont limitées par la petite taille de leurs échantillons et par le manque de précision de leurs évaluations de l’exposition. Nous nous sommes penchés sur la relation entre l’exposition professionnelle à l’amiante et le risque de cancer du rein pour une gamme d’emplois dans une vaste étude populationnelle cas/témoins menée au Canada.

**Méthode:**

Nos données proviennent de la composante cas/témoins du Système national de surveillance accrue du cancer, une étude menée entre 1994 et 1997 dans huit provinces canadiennes. Des hommes atteints d’un cancer du rein confirmé par analyse histologique et des témoins ont rempli un questionnaire sur leur profil sociodémographique et anthropométrique et sur leur régime, leur tabagisme, leur exposition à la fumée secondaire et leur activité physique. Les antécédents professionnels des participants ont aussi été recueillis, notamment chaque emploi occupé pendant au moins un an depuis l’âge de 18 ans. Des hygiénistes professionnels ont assigné à l’aveugle à chaque cas et témoin une exposition à l’amiante en tenant compte de l’intensité, de la fréquence et de la probabilité d’exposition (selon des barèmes de 3 points chacun). Par régression logistique, nous avons estimé la probabilité de cancer du rein (définie à l’aide de trois mesures) chez les participants exposés et les participants non exposés à l’amiante.

**Résultats:**

Les analyses ont porté sur 712 cas et 2454 témoins. Chez les travailleurs ayant déjà été exposés à l’amiante, la probabilité de cancer du rein était supérieure 20 % à celle des travailleurs non exposés (RC de 1,2, intervalle de confiance de 95 % 1,0–1,4 en incluant les travailleurs possiblement exposés). Une légère augmentation du risque a été observée avec une exposition cumulée, et l’intensité de l’exposition était liée à une probabilité accrue de cancer du rein.

**Conclusions:**

Notre étude a trouvé des preuves d’une association entre l’exposition professionnelle à l’amiante et le cancer du rein. L’intensité de l’exposition à l’amiante présentait la relation la plus forte avec le risque de cancer du rein.

## Introduction

Kidney cancer is the fifth most common cancer among Canadian men (2017), and it occurs at double the incidence in men compared to women (22.3 versus 11.3 cases per 100,000 per year) (Committee, C. C. S. S. Canadian Cancer Statistics 2017). Established risk factors for kidney cancer include cigarette smoking, cystic kidney disease, and features of the metabolic syndrome, which include obesity and hypertension (Kabaria et al. 2016). There has long been interest in identifying occupational causes of kidney cancer, but the only established workplace risk factor is trichloroethylene (International Agency for Research on Cancer 2012). Preliminary evidence linking asbestos exposure to increased risk of kidney cancer emerged in the late 1970s; however, since kidney cancer is relatively rare, there have been few large-scale studies of occupational risk factors (Selikoff et al. 1979; Enterline et al. 1987). Asbestos is a commercial term describing six fibrous silicate minerals. Asbestos is a known cause of lung cancer, mesothelioma, and laryngeal and ovarian cancers (IARC Monographs on the Evaluation of Carcinogenic Risks to Humans 2009).

The largest study of kidney cancer available with information included on asbestos exposure was a multicentre case-control study in the mid-1990s (1700 cases, 2300 controls across five countries); a statistically significant 40% increased odds of kidney cancer was found among workers with self-reported ever-exposure to asbestos (Mandel et al. 1995). A meta-analysis conducted in 2000 concluded that there was limited evidence for an association between asbestos exposure and kidney cancer, except possibly among those workers with the highest exposure levels (Sali & Boffetta 2000). However, this meta-analysis included 26 mortality studies and only 6 incidence studies, which limited the ability to distinguish exposures related to disease etiology from factors influencing prognosis. Many of the cohort studies and some case-control studies were further limited by a small number of kidney cancer cases, and the use of crude exposure assessment methods (i.e., self-reported ever-exposure), particularly in case-control studies. Many of the early studies were also industry-specific, which limits the range of exposure levels and broad applicability of the results. Only two studies have examined the relationship between asbestos exposure and kidney cancer since 2000; one found an elevated but not statistically significant odds ratio (Parent et al. 2000), the other found a large elevated risk, but with wide confidence intervals (Mattioli et al. 2002). A recent study of cancer risk among welders found a 30% increased risk of kidney cancer (MacLeod et al. 2016).

Due to the relatively small number of population-based studies, and inconsistent results in the published literature, we investigated the relationship between asbestos exposure and kidney cancer in the context of a large, population-based case-control study in Canada (the National Enhanced Cancer Surveillance System, NECSS). The analysis presented here used data from over 700 incident kidney cancer cases and their controls, making it one of the largest case-control studies of kidney cancer and occupational exposures available. The aim of this study was to examine whether occupational asbestos exposure is a risk factor for kidney cancer in Canadian men.

## Methods

### Study population

Data were drawn from the NECSS case-control study, which was conducted from 1994 to 1997 in eight Canadian provinces. This study has been described previously (Johnson et al. 1998; Villeneuve et al. 2012; Villeneuve et al. 1999) but will be described here in brief. The goal of the NECSS was to investigate the environmental and occupational causes of cancer (Johnson et al. 1998). The current analysis was restricted to men since kidney cancer is more common in men, and the very low prevalence of occupational exposure to asbestos among women would hinder analyses. Specifically, a recent survey of Canadian workplace exposures concluded that over 96% of those previously exposed to asbestos at work were men (CAREX Canada. Occupational exposure to asbestos in Canada 2010). Kidney cancer cases were identified by the provincial cancer registries and all diagnoses were histologically confirmed. Population-based cancer-free controls were recruited using health insurance plans in five provinces (Prince Edward Island, Nova Scotia, Manitoba, Saskatchewan, and British Columbia) and random-digit dialing in Newfoundland and Alberta. In Ontario, a stratified random sample was selected from Ministry of Finance data (Villeneuve et al. 1999). Controls were frequency matched on sex and age (± 5 years) to the overall case distribution for 19 cancer sites included in the NECSS. Response rates for male cases and controls were 73% and 63%, respectively.

### Exposure assessment

Cases and controls provided information for each job held for at least 1 year from the time they were 18 years old until the questionnaire completion date. This information included job title, main tasks, type of industry, and period of employment. The assignment of the dimensions of occupational exposure to asbestos used the expert approach—a methodology applied in previous analyses of the NECSS (Villeneuve et al. 2012; Hu et al. 2008a; Hu et al. 2008b). Occupation and industry codes were assigned by an occupational hygienist, blinded to case status, using the Canadian Classification and Dictionary of Occupations, and Standard Industrial Codes. The same occupational hygienist coded three dimensions of exposure, each on a 3-point scale. These included relative intensity of exposure (low, medium, high), frequency of exposure in a normal work week (< 5%, 5–30%, and > 30% of the time), and degree of confidence that the exposure had occurred (possible, probable, definite) (Hu & Ugnat 2005). This exposure assessment method, referred to as the “expert approach”, is highly reliable and desirable in retrospective exposure assessment (WHO 1995). For the intensity of exposure to asbestos, it is difficult to estimate an absolute comparison to number of fibres per volume of air, but it has been previously suggested that the “medium” intensity category corresponded roughly to the exposure limits in Canada in the early 1980s (i.e., 5 fibres per cubic centimetre) (Villeneuve et al. 2012).

Using these exposure estimates for each job, we constructed three metrics to characterize occupational exposure to asbestos: (1) ever/never exposed, (2) highest attained intensity of exposure (high, medium, low), and (3) a cumulative measure of exposure. The latter metric was defined as the sum across all jobs of intensity multiplied by frequency and duration, as follows:

$$ \mathrm{CE}=\sum \limits_{i=1}^k{C}_i\times {F}_i\times {D}_i $$

where CE = cumulative exposure; *i* represents the *i*th job held, *k* = total number of jobs held, *C* = intensity of asbestos exposure (1 = low, 2 = medium, 3 = high), *F* = frequency of exposure (1 = < 5%, 2 = 6–< 30%, 3 = ≥ 30%), and *D* = duration of employment in years. Descriptive information on the most frequent job held by study subjects was also summarized by job title, most frequent assignment of frequency of exposure, intensity of exposure, and confidence in the coding.

### Statistical and sensitivity analyses

Detailed risk factor information on participants in the NECSS was collected using self-administered questionnaires and included socio-demographic information, anthropometry, diet, smoking and secondhand smoke exposure, and physical activity. Due to differences in the age structure between cases and controls and in data collection methods by province, all analyses were adjusted for age and province. Variables investigated as potential confounders were proxy respondent (since workers themselves might report their work histories more accurately), smoking history (categorical: never smokers, then tertiles of pack-years), secondhand smoke exposure at home and work (categorical: never exposed, then tertiles of smoker-years), body mass index (BMI: categories of normal, overweight, and obese) (Kachuri et al. 2014), income (categorical: low, lower middle, upper middle, high income), physical activity (categorical based on hours per month of moderate or strenuous activity), attained education (also in categories), alcohol consumption (categorical: non-drinkers, then tertiles of drinks/week), and meat consumption (in quartiles). These variables were selected based on previously reported risk factors for kidney cancer (Latifovic et al. 2015; Parent et al. 2007; Siemiatycki et al. 1997).

Unconditional logistic regression was used to estimate odds ratios (OR) and corresponding 95% confidence intervals (CI) between the three asbestos exposure metrics and kidney cancer. The minimally adjusted model included age and province of residence as covariates. A fully adjusted model incorporated kidney cancer risk factors that could also be associated with asbestos exposure (as noted above). Only covariates that produced an appreciable change in the risk estimate (> 10%) were retained. Trend tests were performed by treating the outcome variables as continuous and included the reference group.

Several sensitivity analyses were undertaken to further characterize the observed associations. First, we evaluated how the OR estimates changed when restricting exposures to those classified as probable or definite (which relates to the confidence of the occupational hygienists when assigning exposure). We also examined whether association estimates varied according to kidney cancer histological subtypes (renal cell carcinoma or any other subtype). Finally, to examine the potential impact of latency, we restricted the analysis to men over 40 years of age. The Carleton University Research Ethics Board provided ethics approval for this study.

## Results

There was a total of 727 kidney cancer cases (83% renal cell carcinomas) and 2547 controls initially available for these analyses. After excluding those with missing work histories (i.e., no jobs recorded in the questionnaires), a total of 712 cases and 2454 controls with complete occupational history data were available for analysis. Socio-demographic and health-related characteristics of the study population are presented in Table 1. The mean age of cases was 59, and controls had a mean age of 58. Smoking status and cigarette pack-years were positively, but not statistically significantly associated with kidney cancer, after adjusting for age and province. However, an increase in the odds of kidney cancer was observed in relation to increasing occupational exposure to secondhand smoke. Body mass index corresponding to overweight and obese, as well as high meat intake were positively associated with kidney cancer status, while alcohol intake and physical activity exhibited inverse associations.

**Table 1:** Bivariate relationships between covariates and kidney cancer, adjusted for province and age

Covariates	Cases (*n*, %)	Controls (*n*, %)	Minimally adjusted ORs* (95% CI)
Proxy respondent			
No	464 (65)	1682 (68)	1.0
Yes	248 (35)	780 (32)	1.1 (0.9–1.4)
Pack-years smoking			
0	164 (24)	638 (26)	1.0
> 0–< 10	129 (19)	494 (20)	1.0 (0.8–1.3)
10–< 25	192 (28)	613 (25)	1.1 (0.9–1.4)
25–< 40	114 (16)	353 (15)	1.1 (0.8–1.4)
40+	96 (14)	313 (13)	1.3 (0.9–1.7)
Smoking status at time of interview			
Never smoker	164 (23)	638 (26)	1.0
Former smoker	431 (61)	1300 (53)	1.2 (1.0–1.5)
Current smoker	115 (16)	517 (21)	0.8 (0.6–1.1)
Years since quit smoking^†^			
≥ 31	65 (15)	250 (19)	1.0
21–30	87 (20)	306 (24)	0.9 (0.6–1.4)
11–20	112 (26)	356 (27)	1.0 (0.7–1.5)
≤ 10	167 (39)	387 (30)	1.3 (0.9–2.0)
Occupational secondhand smoke exposure‡			
0	130 (18)	612 (25)	1.0
> 0–< 56 smoker-years	133 (19)	530 (22)	1.3 (1.0–1.7)
56–< 112 smoker-years	157 (22)	477 (19)	1.4 (1.1–1.8)
112–< 191 smoker-years	159 (22)	411 (17)	1.4 (1.1–1.9)
191+ smoker-years	133 (19)	423 (17)	1.4 (1.1–1.9)
Body mass index (kg/m^2^)			
< 25 (normal)	165 (23)	970 (39)	1.0
25–< 30 (overweight)	363 (51)	1128 (46)	1.8 (1.5–2.2)
30+ (obese)	184 (26)	364 (15)	2.9 (2.2–3.7)
Educational attainment			
Less than high school	327 (47)	1026 (42)	1.0
High school complete	134 (19)	423 (17)	0.92 (0.71–1.2)
At least some college	96 (14)	313 (13)	0.98 (0.74–1.3)
At least some university	145 (21)	661 (27)	0.61 (0.48–0.77)
Income adequacy^§^			
High	142 (20)	441 (18)	1.0
Upper middle	209 (29)	663 (27)	1.1 (0.82–1.4)
Lower middle	130 (18)	432 (18)	1.2 (0.90–1.6)
Low	88 (12)	376 (15)	1.0 (0.71–1.3)
Prefers not to answer	143 (20)	550 (22)	1.0 (0.74–1.3)
Physical activity (mean hours/week of moderate or strenuous exercise)^||^			
0	295 (41)	965 (39)	0.83 (0.49–1.4)
> 0–< 10	141 (20)	492 (20)	0.93 (0.70–1.2)
10–< 30	145 (20)	600 (24)	0.74 (0.56–0.98)
30+	131 (18)	405 (16)	1.0
Alcohol intake (mean drinks per week)^¶^			
0	211 (30)	677 (28)	1.0
> 0–< 3	151 (21)	499 (20)	0.96 (0.75–1.2)
3–<8.5	182 (26)	655 (27)	0.82 (0.65–1.0)
8.5+	168 (24)	631 (26)	0.76 (0.60–0.96)
Total meat intake (servings per week)**			
Low (< 5)	161 (23)	682 (28)	1.0
Low-Medium (5–< 8)	152 (21)	555 (23)	1.3 (0.93–1.6)
Medium-high (8–< 12)	174 (24)	568 (23)	1.3 (0.99–1.6)
High (12+)	225 (26)	657 (27)	1.5 (1.2–1.9)

*Odds ratios, adjusted for province and age

†Also adjusted for pack-years smoking, and does not include current or never smokers

‡Number of people smoking near the subject at work multiplied by years of exposure, in quartiles

§Low: income less than $20,000/year, or income $20,000–$29,999 and 4+ people living in the home. Lower middle: income $20,000–$29,999 and less than 4 people in the home, or income $30,000–$39,999 and 4+ people in the home. Upper middle: income $30,000–$39,999 and less than 4 people in the home, or income $40,000–$49,999 and 4+ people in the home. High: income $50,000–$99,999 and less than 4 people in the home or income ≥ $100,000/year

||Moderate exercise includes walking, bowling, dancing, golfing, yardwork, home exercises. Strenuous exercise includes cycling, swimming, tennis, jogging, skiing, and other strenuous exercise

¶Categories represent tertiles

**Defined as quartiles of average number of meat servings per week among the controls

Overall, study subjects held 11,974 jobs over their lifetimes; of these, a total of 655 were coded as having probable or definite asbestos exposure. A further 1275 were coded as possibly exposed. The most common asbestos-exposed jobs were construction workers, mechanics and fabricating workers, and stationary engine and utilities workers (Table 2). Firefighters were most commonly classified as having definite exposure, and in all job categories, most jobs were exposed at medium frequency and low intensity level. Given the small number of workers who had high intensity of asbestos exposure (2 and 3 subjects when excluding or including possible exposure, respectively), these categories were combined.

**Table 2:** Most frequent occupation titles among the 655 jobs with probable or definite asbestos exposure among male kidney cancer cases and controls

		Most common exposure coding*			
Description	Job code (CCDO)^†^	Number of jobs	Confidence	Frequency	Intensity
Construction	8700	231	Probable	Medium	Low
Mechanics, repair, fabrication	8500	198	Probable	Medium	Low
Stationary engine and utilities	9500	109	Probable	Medium	Low
Water transportation	9100	41	Probable	Medium	Low
Firefighters	6100	37	Definite	Medium	Low
Miscellaneous		39	Probable	Medium	Low
Total		655			

*Defined by highest percentage

†Canadian classification and dictionary of occupations

Results for the minimally and fully adjusted models for the three asbestos exposure metrics are shown in Table 3, before and after excluding participants with possible exposure, respectively. In the minimally adjusted models adjusted for age and province, ever exposure to asbestos was associated with increased odds of kidney cancer. Exclusion of subjects with possible exposure results in a slight increase in the association estimate. A monotonic increase in odds of kidney cancer was observed in the models that used the “highest attained exposure to asbestos” metric (Table 3), and again the magnitude of the association increased when excluding the possibly exposed. Trend tests for the relationship between higher attained asbestos intensity and kidney cancer were statistically significant, though this is likely influenced by the inclusion of the reference group in the test. Although all levels of the cumulative exposure metric had ORs above 1, the increase in the odds of kidney cancer was non-monotonic. The test for trend for cumulative exposure was marginally significant when including those with possible exposure (Table 3).

**Table 3:** Adjusted odds ratios of kidney cancer in relation to occupational asbestos exposure

Occupational asbestos exposure	Cases	Controls	Minimally adjusted odds ratios* (95% CI)	Fully adjusted odds ratios† (95% CI)
a) Including all participants, for any level of confidence of exposure to asbestos				
Ever exposed				
Unexposed	445	1679	1.0	1.0
Ever exposed	267	783	1.3 (1.1–1.6)	1.2 (1.0–1.4)
Highest attained exposure				
Unexposed	445	1679	1.0	1.0
Low	251	750	1.3 (1.1–1.6)	1.2 (1.0–1.4)
Medium/high	16	33	1.8 (0.9–3.4)	1.4 (0.7–2.7)
*p* value for trend			*0.002*	*0.027*
Cumulative categories of exposure^±^				
Unexposed	445	1679	1.0	1.0
Low (> 0–< 6)	83	214	1.4 (1.1–1.9)	1.3 (1.0–1.8)
Medium (6–< 16)	85	281	1.2 (0.9–1.6)	1.1 (0.8–1.5)
High (≥ 16)	97	271	1.4 (1.1–1.8))	1.2 (0.9–1.6)
*p* value for trend			*0.008*	*0.066*
b) Restricting to participants with probable or definite exposure to asbestos				
Ever exposed				
Unexposed	445	1679	1.0	1.0
Ever exposed	99	267	1.4 (1.1–1.8)	1.3 (1.0–1.6)
Highest attained exposure				
Unexposed	445	1679	1.0	1.0
Low	84	241	1.3 (1.0–1.7)	1.2 (0.9–1.6)
Medium/high	15	26	2.1 (1.0–4.0)	1.6 (0.8–3.4)
*p* value for trend			*0.01*	*0.07*
Cumulative categories of exposure^±^				
Unexposed	445	1679	1.0	1.0
Low (0–< 10)	30	86	1.4 (0.9–2.2)	1.2 (0.8–2.0)
Medium (10–< 24)	30	82	1.3 (0.8–2.0)	1.2 (0.8–1.9)
High (≥ 24)	38	94	1.5 (1.0–2.2)	1.2 (0.8–1.9)
*p* value for trend			*0.02*	*0.16*

*Adjusted for age and province only

†Adjusted for age, province, body mass index, pack-years of smoking, and education

^±^Cumulative exposure = frequency × intensity × duration of exposure

The final models were additionally adjusted for body mass index, pack-years of smoking, and education, which slightly attenuated the association estimates between asbestos exposure and kidney cancer. Adjustment for smoking did not appreciably change the OR estimates for any of the asbestos metrics. No other confounders considered had a meaningful impact on the relationship between asbestos exposure and kidney cancer risk.

Ever-exposure to asbestos was associated with a 20% increased odds of kidney cancer compared to those who were never exposed, and this was consistent across the models that included or excluded those with possible exposure (Table 3). The positive association between highest asbestos intensity experienced at work remained in the fully adjusted models as well. For cumulative exposure in the fully adjusted models, a statistically significant 40% increased odds of kidney cancer was found in those with low cumulative exposure compared to those without asbestos exposure, but only when observations with possible exposure were included (Table 3).

Only marginal differences were observed when analyses were restricted to renal cell carcinoma, which comprised 83% of all cases. For the “highest attained exposure” to asbestos in the fully adjusted model, excluding possibly exposed workers and non-renal cell carcinoma cases, there was a 50% increased odds of kidney cancer in those who had ever had moderate or high exposure to asbestos compared to those with no asbestos exposure (OR 1.5, 95%CI 0.8–3.2). Restricting the analysis to those over 40 years of age (97% of cases, 84% of controls) did not change our interpretation of the results (OR = 1.3 for highest attained asbestos exposure (95%CI 0.6–2.5), compared to OR = 1.4 when all ages were included).

## Discussion

The results presented here provide some evidence of a relationship between occupational exposure to asbestos and kidney cancer. This adds to the limited and mixed literature surrounding occupational asbestos exposure and kidney cancer risk. Those with higher intensity of exposure to asbestos had increased odds of kidney cancer. Cumulative occupational exposure to asbestos also showed some association with kidney cancer; however, the magnitude of the increased risk was largest in the low cumulative exposure group. This lack of a consistent exposure-response relationship may be due to non-differential misclassification of exposure in ordinal variables with several exposure levels (Schisterman et al. 2009). We controlled for active smoking in our models, though there was not a strong relationship between smoking and the odds of kidney cancer (possibly due to the higher proportion of smokers in our control group). The direction of the effect was as expected, however, and there is evidence in the literature that the relationship between smoking and kidney cancer is confounded by other lifestyle factors, and that cigarette smoke is not as potent of a carcinogen at this site (Birkett 1992).

The kidneys are not in direct contact with asbestos through inhalation, but clearance from the lungs may lead to translocation to the kidneys where the fibres have the opportunity to interact with tissue and initiate carcinogenesis (Choi et al. 2010). We know this process can take place with asbestos, as it is a known cause of ovarian cancer, another site with no direct lung contact (IARC Monographs on the Evaluation of Carcinogenic Risks to Humans 2009).

Previous results on this topic have been mixed for several reasons. These include focus on a specific industry, lack of information on confounders, small sample sizes, and less-detailed exposure assessment. In the current study, we were able to more fully overcome these limitations with our large population-based sample, detailed information on confounders (including smoking), and a detailed occupational history and exposure estimation.

The previously mentioned meta-analysis (2000) of the relationship between asbestos exposure and kidney cancer found it “unlikely that asbestos exposure is responsible for an important increase in kidney cancer risk; however, high asbestos exposure might entail a small increase in risk” (Sali & Boffetta 2000). This is consistent with the results of our study, which was based on a larger sample size. However, there were important differences between our study and this meta-analysis (Sali & Boffetta 2000), which included kidney cancer mortality as the endpoint and had a limited sample size of 69 incident cancers.

In a more recent analysis of occupational asbestos exposure and kidney cancer risk, a hospital-based case-control study found sevenfold odds of renal cell carcinoma for male asbestos-exposed workers, as well as an increased risk among railway workers (Mattioli et al. 2002). However, the exposure assessment in that study, while complete for the working life, only included a broad job classification and crude asbestos exposure assessment, and so was less detailed than our study.

Restricting the analyses to include only those with probable or definite asbestos exposure increased the magnitude of some association estimates, which would be expected due to higher certainty in exposure status. However, this was partially offset by the attenuated sample size after the restriction was applied (Teschke et al. 2002). Similar association estimates were observed in Tables 3 (less restrictive definition of exposure) and 3b (more restrictive), suggesting that those jobs flagged as possibly exposed were more similar to the probable/definite exposed jobs than not. The job coding methodology instructs coders to default to the “possible” category when detailed task information is not available.

There were minimal differences in risk estimates when restricting analyses to renal cell carcinoma cases only, but this is likely because 83% of cases had renal cell carcinoma. We also did not see a difference in the analysis when participants under the age of 40 were excluded, so we opted to leave them in the analysis, with the understanding that the latency between asbestos exposure and kidney cancer is unknown as of yet.

This study has several strengths, including a lifetime occupational history, which allowed us to create several metrics of asbestos exposure. The NECSS also contains a large amount of detailed information on personal and other risk factors for cancer, and we were thus able to consider important potential confounders for the relationship between occupational exposure and kidney cancer. There is some risk of recall bias in the confounding variables from the retrospective nature of data collection inherent to the NECSS. However, this is unlikely to be a problem for the assessment of asbestos exposure, as this was assigned by occupational hygienists (though we cannot assess whether cases were more careful in their documentation of the jobs held). The participation rates for kidney cancer cases (73% among men) and controls (63% among men) are both typical for a population-based study like the NECSS, but there is the potential that participation bias may have affected our results. If, for example, wealthier men were likelier to agree to be a control (which is expected), then the case series could have important differences from the controls that are not based on true distributions in the population. Although we were able to account for most of the relevant confounders, information on exposure to trichloroethylene was not available, as task information was not sufficiently detailed to assess it. However, although this is an established kidney cancer risk factor, we expect very low prevalence of exposure in our population. While exposure assessment was carried out by a single expert, we observed very high inter-rater assessment of asbestos exposure in our previous NECSS-based study of lung cancer applying the same methodology (Villeneuve et al. 2012).

The main strength of this study was the detailed exposure assessment approach. Reports of job histories have been shown to be valid (Baumgarten et al. 1983). While the lack of direct measurement of asbestos exposure is a limitation, collecting such measures is not feasible in most large-scale, population-based studies, especially case-control designs. Therefore, expert assessment by occupational hygienists is considered to be the reference approach for epidemiological studies such as this one (Bouyer & Hémon 1993). Furthermore, our expert-based exposure assessment methodology has high reliability, as documented in previously published case-control studies that have employed this method (WHO 1995; Fritschi et al. 1996). Additionally, the occupational hygienists were blinded to case status while coding the jobs, so any misclassification of exposure would be non-differential, and thus unlikely to be a source of bias. Our detailed consideration of reliability scores presents a more fulsome picture of the quality of the exposure assessment.

## Conclusion

In this large, population-based study of Canadian men, we found some evidence of a relationship between occupational exposure to asbestos and kidney cancer risk. The association was more pronounced for workers exposed at higher intensity.
